# Entrepreneurial Capability, Career Development, and Entrepreneurial Intention: Evidence From China's HR Survey Data

**DOI:** 10.3389/fpsyg.2022.870706

**Published:** 2022-04-11

**Authors:** Wenxin Hu, Hua Liu, Yuqin Tian, Xiaohong Zhang, Yufei Mao

**Affiliations:** ^1^Enterprise Development and Management Innovation Research Center, School of Economics and Management, Beijing Institute of Petrochemical Technology, Beijing, China; ^2^Business School, Hebei Normal University, Shijiazhuang, China; ^3^School of Economics and Management, Beijing Institute of Petrochemical Technology, Beijing, China; ^4^Enterprise Development and Management Innovation Research Center, School of Economics and Management, Beijing Institute of Petrochemical Technology, Beijing Great Wall Scholar, Beijing, China; ^5^School of Labor Economics, Capital University of Economics and Business, Beijing, China

**Keywords:** entrepreneurship, entrepreneurial ability, career development, entrepreneurial intention, HR staff

## Abstract

Based on 2018 China's Human Resource Employees Survey Data, this study uses the probit model to examine the impact of entrepreneurial ability and career development on HR's entrepreneurial intention. In terms of entrepreneurial ability, the results show that the educational background of Human Resource Management, cross-disciplinary knowledge, job-hopping experience, and the number of subordinates have significant positive impacts on HR's entrepreneurial intention. In terms of career development, lack of promotion space, skill upgrading opportunities, and lower than expected income have significant positive impacts on HR's entrepreneurial intention, and these impacts are heterogeneous among different enterprises. This study suggests that potential entrepreneurs can be identified from the explicit characteristics, which reflect HR's entrepreneurial abilities, and it is necessary to face up to the influence of career development on HR's entrepreneurial intention and encourage them to participate in on-the-job entrepreneurship. This study suggests that HR's entrepreneurial ability should be regarded as an important starting point for entrepreneurial success, and it is necessary to improve HR's career development system to create more opportunities for on-the-job entrepreneurship, and government should implement differentiated and precise entrepreneurial support policies to encourage HR's entrepreneurship.

## Introduction

With the rapid development of the digital economy and the introduction of entrepreneurial policies, entrepreneurship has become a key topic at present (Wu et al., 2019; Yuan et al., 2020; Wang et al., 2021; Scuotto et al., 2022). In the era of the knowledge economy, the human resource service industry plays an important role in promoting economic development and job creation (Chen and Zhou, 2017; Zhao et al., 2019). Actively guiding entrepreneurship in the field of human resource services not only caters to the market demand for high-quality talent services but also helps to stimulate the vitality of market subjects and create new jobs (Wu, 2016). According to the latest statistical report on the development of human resource services issued by the Ministry of Human Resources and Social Security, China, has set up 39,600 human resource service agencies in 2019, which employed 675,000 people and generated a revenue of 1.96 trillion yuan annually. As an important participant among enterprise leaders, HR not only possesses professional knowledge and rich practical experience but is also familiar with the policies and regulations in this field and has some understanding of the future development trend of the industry. Therefore, encouraging HR to participate in entrepreneurship is of great practical significance to HR's career development, as well as the innovation and entrepreneurship of the human resource service industry. As for the HR group, which kind of abilities and characteristics reveal more entrepreneurial motivation? Whether career development will affect HR's entrepreneurial intention? Whether these impacts are heterogeneous in different enterprises? This study carries out further discussion and research on the questions mentioned above.

Entrepreneurship is an important topic in the field of economic management research (Yin et al., 2015; Karayol and Taze, 2020; Civera et al., 2021; Deng et al., 2021). As for the influencing factors of entrepreneurship, economic studies mainly focus on variables such as individual characteristics, family characteristics, and macro policies (Wang and Zhang, 2017), while management studies concentrate on analyzing the relationship between organizational system, enterprise culture, and entrepreneurship (Li and Li, 2009; Tang and Tang, 2012); psychological research focuses on exploring psychological characteristic variables such as entrepreneurial passion, self-efficacy, and risk perception (Clercq et al., 2013; Fang and Zhang, 2016). In recent years, the study of Human Resource Management (HRM) and entrepreneurship has gradually attracted extensive attention for scholars (Liu and Wang, 2011). Among HRM factors, which influence entrepreneurial behaviors, entrepreneurial abilities were regarded as the comprehensive knowledge and skills, which a successful entrepreneur needs to possess, and they played an important role in promoting entrepreneurial behaviors (Mitchelmore and Rowley, 2013; Bayon et al., 2015). This study focuses on the HR staff, and it attempts to reflect their entrepreneurial abilities from the aspect of educational background, job-hopping experience, and interdisciplinary background, and it empirically discusses the impact of entrepreneurial ability on the entrepreneurial intention. Different from the entrepreneurship for college students and migrant workers, the HR staff are generally employed in the enterprises before starting their own business. Their salary, benefits, and future career development may have a significant impact on entrepreneurial intentions (Weng and Xi, 2010). Therefore, it is of practical significance to analyze the influence of career development on entrepreneurial intention.

The contributions of this study are the following aspects: First, different from the research perspective in previous studies in which entrepreneurs are usually studied as a whole group (Deng et al., 2021; Yang et al., 2021), this study focuses on the specific group of HR staff and discusses the influence of HR's entrepreneurial ability and career development on their entrepreneurial intention, which enriches the research in related fields. Second, existing researches on HR's entrepreneurial intention often use qualitative research methods such as literature research, case analysis, and in-depth interviews, but few studies explored the quantitative methods (Chen et al., 2021). Based on 2,219 samples of HR staff, this study builds an econometric model and empirically tests the influence of entrepreneurial ability and career development on HR's entrepreneurial intention. Third, existing literature considered entrepreneurs as the homogeneous groups, in fact, for HR who participate in on-the-job entrepreneurship and the influencing factors of their entrepreneurial intentions will be heterogeneous in different enterprises. In view of this, this study conducts sample analysis according to the enterprises' size, type, and city levels, and it investigates the heterogeneity of influencing factors of entrepreneurial intention in different enterprises.

## Literature Review and Hypotheses

### Entrepreneurial Capability and Entrepreneurial Intention

Entrepreneurial capability, defined as the entrepreneurs' special skill or experience, helps entrepreneurs seize business opportunities, leads the entrepreneurial team, and creates business value to successfully achieve the entrepreneurial goals (Xie and Huang, 2014; Ge and Zhao, 2021). As for the measurement of entrepreneurial capability, the existing literature mainly measured individual entrepreneurial ability through organizational ability, strategic ability, and other subjective indicators, and fewer objective indicators were selected to describe entrepreneurial ability (Lans et al., 2010). In fact, subjective self-assessment of entrepreneurial capability may exaggerate the contribution of entrepreneurial capability to the entrepreneurial intention (Su and Duan, 2015). In order to reduce the measurement error brought by the subjective evaluation index, this study attempts to select the objective proxy variables, which reflect the entrepreneurial capability, and tries to test the impact of entrepreneurial capability on entrepreneurial intention. In particular, for the HR staff concerned in our study, due to their work content in the field of HRM, the level of HR professional knowledge, the ability to manage organizational teams, and their job-hopping experience may affect HR's accurate assessment of entrepreneurial activity (Qian, 2007).

Therefore, our study will measure the entrepreneurial capability from the aspects of HR's educational background, cross-disciplinary background, job-hopping experience, and number of subordinates. Among them, if an HR has an education background in HRM, he/she will be able to more easily combine theory and practice and seize entrepreneurial opportunities. If an HR has a cross-disciplinary education background, the HR will have a cross-disciplinary way of thinking, and it is conducive to the creation of their entrepreneurial ideas (Edward, 2005). If an HR has job-hopping experience, which means that HR has work experience in different companies, and it is conducive to their own entrepreneurial activity. The more subordinates an HR manages in the company, the higher his/her entrepreneurial intention will be. The number of subordinates not only represents HR's prominent leadership and management capabilities but also indirectly reflects the higher management positions; this advantage will help them to obtain more social financing support and then promote the entrepreneurial intention. Based on the above analysis, it can be seen from the educational background, cross-discipline, job-hopping, and the number of subordinates can comprehensively reflect the entrepreneurial capability, which may have a positive impact on the individual entrepreneurial intention. Therefore, this study proposes the following hypotheses:

**Hypothesis 1:** If an HR has an education background in HRM, his/her entrepreneurial intention will be higher.
**Hypothesis 2:** If an HR has a cross-disciplinary education background, his/her entrepreneurial intention will be higher.
**Hypothesis 3:** If an HR has job-hopping experience, his/her entrepreneurial intention will be higher.
**Hypothesis 4:** The more subordinates an HR manages in the company, the higher his/her entrepreneurial intention will be.

### Career Development and Entrepreneurial Intention

Entrepreneurial intention involves not only the entrepreneurial ability but also career development. In order to achieve rapid career growth, individuals could choose to create a new company, and it was an important way to achieve one's career goals (Weng and Xi, 2010; Adedoyin, 2021; Trabskaia and Mets, 2021). In the existing research on entrepreneurship, researchers mostly divided entrepreneurial forms into survival entrepreneurship and opportunistic entrepreneurship (Yue, 2014), and the influencing factors of these two entrepreneurial forms are also different. Among them, survival entrepreneurship generally refers to the lack of better job options and chooses to start a business in order to satisfy the individual's basic living conditions, and their entrepreneurial success depends mainly on personal qualifications, start-up costs, and policy support. Opportunistic entrepreneurship refers to the pursuit of career development and undertaking entrepreneurship, which are mainly influenced by such factors as entrepreneurial risk, resource availability, and career development (Yue, 2014). According to the above classification, the HR staff concerned in our article mainly belong to opportunistic entrepreneurs. Therefore, in order to analyze their entrepreneurial intention, it is important to pay attention to the influence factor of career development. In addition, most entrepreneurs in this study focus on college students, migrant workers, and unemployed people. Most of them are employed for the first time or flexibly and are rarely subjected to the existing organizations when they establish the enterprises. For the HR staff studied in this study, when choosing to start a business, they will consider whether the compensation and benefits offered by the current companies can satisfy their long-term career development.

Therefore, this study attempts to examine the impact of career development on the individual entrepreneurial intention from three aspects: position promotion space, skills upgrading space, and expectation income. Among them, if individuals can't achieve rapid promotion or unable to obtain obvious skills upgrading, the cost of quitting their current jobs is correspondingly low (Weng and Xi, 2010), and it will increase their entrepreneurial intentions. Relative income mainly examines the gap between an individual's actual income and expected income. If the actual income is lower than expected, an individual will have a relatively high willingness to quit and start a business (Kautonen et al., 2017). Based on the above analysis, this study proposes the following hypotheses:

**Hypothesis 5:** If an HR has a larger promotion space in the current organization, his/her entrepreneurial intention will be reduced.
**Hypothesis 6:** If an HR has more space to improve skills in the current organization, his/her entrepreneurial intention will be reduced.
**Hypothesis 7:** If an HR obtains the actual income, which is higher than the expected value, his/her entrepreneurial intention will be reduced.

## Data and Methods

### Data

The data we used in this study come from 2018 China's Human Resource Employees Survey, which was sponsored by the School of Labor and Human Resources, Renmin University of China. The data samples were from the HR staff in China, the total samples in the database was 5,303, and the effective samples were 2,219 when we finished data cleaning. The HR staff were employed in various types of institutions and enterprises, which involved government departments, state-owned enterprises, foreign-funded enterprises, private enterprises, and joint-venture enterprises, and it effectively ensured the research representations. The questionnaire contained personal information, career development, and HR management status. It also involved variables of entrepreneurial intentions as shown in Table 1.

**Table 1:** Variables and descriptions.

**Dimension**	**Variables**	**Variables descriptions**
Entrepreneurial variables	Entrepreneurial intention	Have a willingness to start a business (Yes = 1, No = 0)
Entrepreneurial capability	Human Resource Management background	Have an education background of Human Resource Management (Yes = 1, No = 0)
	Cross-discipline background	Have two or more learning backgrounds in different disciplines (Yes = 1, No = 0)
	Job-hopping experience	The numbers of job- hopping from graduation to current work
	Number of subordinates	None people =1, 1–5 people = 2, 6–10 people = 3, 11–20 people = 4, 21–50 people = 5, 50 and above people = 7
Career development	Position promotion	Have the promotion space in this company (very little = 1, very much = 5)
	Skills upgrading	The space for skills upgrading in this company (very little = 1, very much = 5)
	Relative income	Relative comparison between real income and expected income (lower than = 1, be equal to = 2, more than = 3)
Personal characteristics	Gender	Male = 1, Female = 0
	Age	The actual age calculated according to the year of investigation (Unit: year)
	Marital status	Married = 1,Unmarried/single = 0
	Political status	Party member = 1, Other = 0
	Years of education	Actual number of years of education (Unit: year)
Social capital	Colleagues	The number of colleagues with good relationships within a company
	Colleagues' positions	None = 1, Ordinary staff = 2, First-line manger = 3, Middle manager = 4, Top manager = 5
	External friend	The number of friends with good relationships outside the company
	Friend's position	None = 1, Ordinary staff = 2, First-line manger = 3, Middle manager = 4, Top manager = 5
Psychological characteristics	Openness	like to try new things (Relative disagree = 1, Relative agree = 5)
	Emotional stability	Have the relaxed mood and be good at relieving pressure (Relative disagree = 1, Relative agree = 5)
	Self-efficacy	Believe that having the abilities to accomplish the tasks (Relative disagree = 1, Relative agree = 5)
Occupational value	External remuneration	Believe that career success is a high external reward (Relative disagree = 1, Relative agree = 5)
	Internal satisfaction	Believe that career success is achieved through hard work (Relative disagree = 1, Relative agree = 5)
Enterprise characteristics	Enterprise scales	Micro enterprise = 1, Small enterprise = 2, Medium enterprise = 3, Large enterprise = 4
	Enterprise types	Government/institutions = 1, State-owned enterprise = 2, Private enterprises = 3, Foreign/joint-ventures enterprises = 4
	City levels	The first-tier city = 1, The new first-tier city = 2, The second-tier city = 3,The third-tier city = 4

#### Explained Variables: Entrepreneurial Intention

Whether HR has an entrepreneurial intention is the primary motive for future entrepreneurial activities (Shabnaz and Islam, 2021). In the analysis, the entrepreneurial intention is reflected in the questionnaire of “whether individual has the entrepreneurial ideas,” and it is measured by the two-value variable. Entrepreneurial action is reflected in the “practical entrepreneurial action preparation.” Among them, 1 represents the willingness to start a business, and 0 represents the lack of willingness to start a business.

#### Explanatory Variables: Entrepreneurial Ability and Career Development

Among them, the entrepreneurial ability is mainly measured by the educational background of HRM, educational background of cross-discipline, job-hopping experience, and the number of subordinates, while career development is mainly measured by job promotion, skills upgrading, and relative income. In addition, from the perspective of other influencing factors of entrepreneurial intention, existing studies have also focused on the impact of demographic characteristics, social network, Big Five personality, and entrepreneurial environment (Lin and Jiang, 2012; Bing et al., 2015). In this study, factors such as personal characteristics, social capital, psychological characteristics, and occupational value were controlled in the regression model. Personal characteristics include gender, age, marital status, political status, and education. Social capital is mainly examined from the perspective of the number of colleagues or friends inside and outside the organization, which reflects the breadth and depth of the personal social capital, respectively. Psychological characteristics are measured by openness and emotional stability in the Big Five personality and self-efficacy in psychological capital. Occupational value is mainly measured from the individual's evaluation of external reward and internal satisfaction for occupational success.

### Model Design

The probit model is conducted to analyze the impact of entrepreneurial capability and development space on the entrepreneurial intention:


(1) $$\begin{array}{l} \mathrm{Pr}obit(EI=1)=\Phi (\alpha +\beta competence+\gamma development\\ \;+\lambda X_{i}) \end{array}$$

In Formula 1, *EI* represents HR's entrepreneurial intention, the core explanatory variable *competence* represents the entrepreneurial capability, the *development* represents the career development space, and *X*_*i*_ represents the other explanatory variables, such as personal characteristics, social capital, psychological characteristics, occupational values, and enterprise characteristics. β, γ, and λ, respectively, indicate the coefficient values that need to be estimated.

Based on Formula 1, we also focus on the impact of individual entrepreneurial capability and development space in different enterprises. According to enterprise scales, enterprise ownerships, and city levels, we carry out sample size regression and analyze the heterogeneity of influencing factors of HR entrepreneurial intention in different enterprises.

### Descriptive Statistics

Table 2 shows the descriptive statistical results. After eliminating the missing key variables, 2,219 samples are obtained. Among them, the sample who has entrepreneurial intention is 1,137, accounting for 51.2%, and the sample of lacking entrepreneurial intention is 1,082, accounting for 48.8%. From the perspective of core explanatory variables, 52.7% of HR staff have the educational background of HRM, 19.9% of HR staff have the educational background of cross-discipline, and their average experience of job-hopping is 1.2 times. The *t*-test results show that the HR who has entrepreneurial intention with a significant proportion of cross-disciplinary background, and their experience of changing work and the number of subordinates are relatively large. From the perspective of career development, the promotion space, skill upgrading space, and relative income status of HR with entrepreneurial intention are significantly lower than those of HR without entrepreneurial intention.

**Table 2:** Descriptive statistics of the main variables.

**Dimension**	**Variables**	**Whole** ** sample**	**Have** **entrepreneurial intention**	**Have** **not entrepreneurial intention**	***T*** **-test**
Entrepreneurial capability	Human Resource Management background	0.527 (0.499)	0.538 (0.499)	0.516 (0.500)	0.022 (0.011)
	Cross-discipline background	0.199 (0.399)	0.237 (0.426)	0.159 (0.366)	0.078^***^ (0.002)
	Job-hopping experience	1.236 (1.365)	1.450 (1.425)	1.010 (1.260)	0.440^***^ (0.014)
	Number of subordinates	1.073 (1.378)	1.235 (1.444)	0.903 (1.284)	0.332^***^ (0.018)
Career development	Position promotion	2.862 (0.952)	2.799 (0.943)	2.927 (0.957)	−0.127^***^ (0.009)
	Skills upgrading	3.194 (0.940)	3.150 (0.953)	3.240 (0.924)	−0.090^***^ (0.002)
	Relative income	1.446 (0.543)	1.395 (0.532)	1.500 (0.55)	−0.105^***^ (0.008)
Personal characteristics	Gender	0.403 (0.491)	0.442 (0.497)	0.362 (0.481)	0.080^***^ (0.013)
	Age	29.47 (6.026)	29.51 (5.906)	29.42 (6.152)	0.093 (0.031)
	Marital status	0.422 (0.494)	0.395 (0.489)	0.451 (0.498)	−0.056 (0.020)
	Political status	0.505 (0.500)	0.536 (0.499)	0.473 (0.513)	0.062^***^ (0.003)
	Years of education	16.69 (1.863)	16.59 (1.925)	16.79 (1.791)	−0.191^**^ (0.012)
Social capital	Colleagues	4.100 (1.662)	4.237 (1.658)	3.957 (1.654)	0.280^***^ (0.010)
	Colleagues' positions	5.503 (1.755)	5.672 (1.667)	5.325 (1.826)	0.347^***^ (0.020)
	External friend	2.067 (0.954)	2.186 (0.961)	1.942 (0.931)	0.244^***^ (0.005)
	Friend's position	2.103 (0.979)	2.274 (0.964)	1.923 (0.963)	0.351^***^ (0.010)
Psychological characteristics	Openness	3.681 (0.738)	3.764 (0.718)	3.594 (0.749)	0.169^***^ (0.006)
	Emotional stability	3.298 (0.813)	3.301 (0.835)	3.295 (0.790)	0.006 (0.001)
	Self-efficacy	4.007 (0.712)	4.050 (0.708)	3.961 (0.712)	0.089^***^ (0.004)
Occupational value	External remuneration	3.371 (1.001)	3.369 (0.983)	3.374 (1.021)	0.005 (0.005)
	Internal satisfaction	3.754 (0.886)	3.812 (0.871)	3.694 (0.898)	0.117^***^ (0.002)
Observations		2.219	1.137	1.082	-

The data in the table are the mean of the sample, and the data in the parenthesis are standard deviations. The symbols ^^***^^, ^**^, and ^*^ are used to indicate significances at the level of 1%, 5% and 10% respectively.

In other explanatory variables, from the perspective of personal characteristics, male account for 40.3%, and their average age is 30 years. Among them, 50.5% are married, and 42.2% are the members of Chinese Communist Party. Their average education year is 16.7 years. From the perspective of social capital, the HR with entrepreneurial intention has more related colleagues and external friends, and these colleagues or friends have relatively high administrative positions. From the perspective of psychology, HR with entrepreneurial intention has a higher score on openness and self-efficacy. From the point of view of career value, the HR with entrepreneurial intention prefers to agree with the concept of value that achieves their career goals through hard work.

## Results

### Basic Empirical Results

Based on the above analysis strategies, this study constructs the probit model and analyzes the impact of entrepreneurial capability and career development on entrepreneurial intention. Table 3 lists the results of marginal effects and robust standard error. In terms of entrepreneurial capability, the estimated coefficients of all the variables are significantly positive in the whole sample estimation results. Among them, the coefficient of cross-disciplinary educational background is 0.125, indicating that after controlling for other factors, HRs with two different academic backgrounds tend to have an entrepreneurial tendency of 12.5% more than that of a single subject background. The empirical results show that H1 is proved. In addition, HRM background, job-hopping experience, and the number of subordinates have a significant positive impact on entrepreneurial intention, indicating that HRs who have the HRM professional background and have the experience of changing jobs and managing more subordinates will have relatively high entrepreneurial intention. The empirical results show that H2, H3, and H4 are proved. Equations (2) and (3) show that there are some gender differences in the empirical results. In the male sample, the marginal effects of cross-disciplinary background and job-hopping factors are significantly positive, indicating that for men, having a cross-disciplinary background and more job-hopping experiences are more likely to start-up a business. In the female sample, the coefficients of each variable are significantly positive; these results reveal that in addition to cross-disciplinary background and job-hopping experience, HRM background and number of subordinates also have a positive effect on women's entrepreneurial intention.

**Table 3:** The impact of entrepreneurial capability and career development on entrepreneurial intention.

**Dimension**	**Variables**	**(1)** **Whole samples**	**(2)** **Male samples**	**(3)** **Female samples**
Entrepreneurial capability	Human Resource Management background	0.046^**^ (0.023)	0.038 (0.036)	0.053^*^ (0.029)
	Cross-discipline background	0.125^***^ (0.026)	0.074^*^ (0.043)	0.152^***^ (0.033)
	Job-hopping experience	0.041^***^ (0.009)	0.047^***^ (0.014)	0.035^***^ (0.012)
	Number of subordinates	0.023^***^ (0.009)	0.011 (0.012)	0.039^***^ (0.012)
Career development	Position promotion	−0.036^***^ (0.013)	−0.039^*^ (0.021)	−0.042^**^ (0.016)
	Skills upgrading	−0.026^**^ (0.013)	−0.031 (0.021)	−0.024 (0.016)
	Relative income	−0.089^***^ (0.019)	−0.09^***^ (0.03)	−0.091^***^ (0.025)
Personal characteristics	Gender	0.085^***^ (0.021)	–	–
	Age	−0.014^***^ (0.002)	−0.015^*^ (0.003)	−0.013^***^ (0.003)
	Political status	−0.003 (0.025)	0.045 (0.041)	−0.025 (0.031)
	Marital status	0.085^***^ (0.025)	0.072^*^ (0.042)	0.091^***^ (0.031)
	Years of education	−0.008 (0.007)	0.001 (0.01)	−0.018^*^ (0.009)
Social capital	Colleagues	0.001 (0.007)	0.003 (0.012)	0.002 (0.009)
	External friend	0.015^**^ (0.007)	0.011 (0.011)	0.014^*^ (0.008)
	Colleagues' positions	0.026^**^ (0.013)	0.012 (0.022)	0.029^*^ (0.017)
	Friend's position	0.06^***^ (0.013)	0.068^***^ (0.022)	0.057^***^ (0.016)
Psychological characteristics	Openness	0.075^***^ (0.015)	0.046^*^ (0.024)	0.093^***^ (0.019)
	Emotional stability	−0.038^***^ (0.013)	−0.013 (0.022)	−0.05^***^ (0.016)
	Self-efficacy	0.015 (0.015)	0.008 (0.024)	0.023 (0.02)
Occupational value	External remuneration	−0.014 (0.011)	0.014 (0.017)	−0.035^**^ (0.014)
	Internal satisfaction	0.031^**^ (0.013)	0.026 (0.019)	0.038^**^ (0.017)
Control variables		Yes	Yes	Yes
Wald χ^2^		255.01^***^	91.46^***^	186.53^***^
Observations		2,219	894	1,325

Note: Marginal effects are reported in the table, and robust standard errors are shown in parentheses. The values with ^***^, ^**^, and ^*^ are significant at the level of 1, 5, and 10%, respectively. The enterprise size, the nature of the enterprise, and the city rank are added to the regression.

Judging from the estimation results of career development, the coefficients of position promotion, skill upgrading, and relative income are all significantly negative in the whole sample estimation. This shows that if HRs have more job or promotion opportunities in the current company, or if they can enhance their skills continuously, and their actual income is higher than their expected income, then their entrepreneurial intention will be reduced accordingly. The empirical results show that H5, H6, and H7 are proved. From the gender regression results, in the male sample, the marginal effects of a job promotion, skill upgrading, and relative income are significantly negative. For women, the coefficient of job promotion and relative income are significantly negative, while the coefficient of skill upgrading is negative but not significant. The regression results show that the lack of position promotion and low relative income can significantly improve the entrepreneurial intention for both male and female HR staff, and the lack of skill upgrading only can improve the male entrepreneurial intention.

For the explanatory variables of personal characteristics, the marginal effects of gender and marital status are significantly positive, which indicates that the entrepreneurial intention of male HR is higher than female HR, and the entrepreneurial intention of married HR is higher than that of unmarried groups. The coefficient of age is significantly negative, indicating that HR will have a lower probability of entrepreneurial intention when their age increases. But the coefficient of political status and educational years are not significant, indicating that whether HR is a party member or not, as well as the level of education, their entrepreneurial intentions are not significant. However, from the direction of the coefficient, the entrepreneurial intentions of male party members and highly educated men are relatively high, while women's willingness to start a business is lower.

In terms of social capital, the effect of colleagues is not significant, indicating that if HR has a relatively good number of colleagues in the current company, it will not affect their entrepreneurial intention. In the full samples and female samples, the coefficient of external friends and colleagues' positions and friends' positions are significantly positive, indicating that for HR especially women HR, the more you have good friends outside your company and the more coworkers or colleagues hold the highest managerial positions, their entrepreneurial intentions will be stronger. This is because the number and position of friends or colleagues outside the company reflect the breadth and depth of individual-owned social capital. On the one hand, having more friends may exchange information in various sectors timely, which is conducive to the discovery of business opportunities. On the other hand, these colleagues or friends who occupy higher management positions may help HR to get more money and facilitate when sharing the entrepreneurial resources. In the male sample, only the coefficient of friends' position is significantly positive. It shows that for male HR, the higher the position of a friend is, the more obvious it will affect their entrepreneurial intentions.

In terms of psychological characteristics, openness is significantly positive in the whole sample, indicating that HR staff who are open-minded and inclined to try new things are more willing to start a business. Emotional stability is significantly negative in the whole sample and the female sample, indicating that HR who is calm and intense has a relatively low willingness to start a business. This is in line with the conclusion of previous studies (Zhao and Seibert, 2006). Compared with general managers, entrepreneurs score higher in openness and conscientiousness, but lower in easy-going and emotional stability. The effect of self-efficacy on entrepreneurial intention is not obvious.

In the aspect of occupational value, the coefficient of internal satisfaction is significantly positive, but the external remuneration coefficient is insignificant. It reveals that the more inclined HR is to agree that achieving career success through hard work, the higher their entrepreneurial intention will be. In terms of gender results, the occupational value variable has no significant effect on male HR staff. In the female samples, however, the external remunerations are significantly negative, but their internal satisfactions are significantly positive, which indicates that the more HR women agreed with the occupational value that “career success comes from fulfilling job aspirations rather than getting high compensation,” the more willing they are to start their own business.

### Heterogeneity Analysis

In order to further explore the heterogeneity results, this study constructs the subsample regression to make clear the impacts of entrepreneurial capability and career development in different enterprises. Based on literature research and empirical results, HR's entrepreneurial ability and career development may be affected by enterprise types, enterprise scales, and city levels. In general, HRs employed in large enterprises and first-tier cities have the higher entrepreneurial ability and more career development opportunities. Given the consideration of heterogeneity analysis, according to the classification of enterprise types, enterprise size, and city levels, we make the sample regression to analyze the influencing factors of entrepreneurial intention. Table 4 lists the influence of entrepreneurial ability and career development on entrepreneurial intention in different enterprise nature. As for the entrepreneurial capability, the marginal effect of HRM background is significantly positive only in the sample of foreign/joint-venture enterprises. Job-hopping is significantly positive in government/institutions, private enterprises, and foreign/joint-ventures enterprises. The number of subordinates has a significant positive impact on entrepreneurial intentions in private and foreign/joint-venture enterprises. In the aspects of career development, promotion in government/institutions and private enterprises is significantly negative, which means that if HR has more promotion space in these two types of enterprises, it will reduce their entrepreneurial intention. The marginal effects of relative income in the sample of private enterprises and foreign/joint venture enterprises are negative, and this shows that if HR's actual income is higher than the expected value in these two types of enterprises, then it will significantly reduce their entrepreneurial intentions.

**Table 4:** Heterogeneous analysis results in different enterprise types.

**Dimension**	**Variables**	**(4) Different enterprise types**			
		**Government/** **institutions**	**State-owned enterprise**	**Private enterprises**	**Foreign/joint-ventures enterprises**
Entrepreneurial capability	Human Resource Management background	0.047 (0.064)	0.005 (0.054)	0.016 (0.034)	0.108^**^ (0.046)
	Cross- discipline background	0.158^**^ (0.071)	0.137^**^ (0.054)	0.086^**^ (0.041)	0.196^***^ (0.056)
	Job-hopping experience	0.061^*^ (0.035)	0.020 (0.023)	0.037^***^ (0.013)	0.047^**^ (0.019)
	Number of subordinates	−0.002 (0.023)	0.013 (0.017)	0.037^***^ (0.014)	0.031^*^ (0.018)
Career development	Position promotion	−0.086^**^ (0.041)	−0.04 (0.028)	−0.04^**^ (0.019)	−0.011 (0.027)
	Skills upgrading	−0.048 (0.037)	−0.007 (0.029)	−0.030 (0.019)	0.0001 (0.027)
	Relative income	−0.075 (0.053)	−0.061 (0.039)	−0.091^***^ (0.029)	−0.09^**^ (0.042)
Control variables		Yes	Yes	Yes	Yes
Wald χ^2^		72.33^***^	64.28^***^	124.06^***^	85.07^***^
Observations		260	521	953	485

Marginal effects are reported in the table, and robust standard errors are shown in parentheses. The values with ^***^, ^**^, and ^*^ are significant at the level of 1, 5, and 10%, respectively. The variables such as personal characteristics, social capital, psychological characteristics, occupational value, and enterprise characteristics are added to the regression. Due to the length limit, this study shows only the estimated results of some variables.

Heterogeneous analysis results for different enterprise sizes are shown in Table 5. As for the impacts of entrepreneurial capability, the marginal effects of HRM background in medium and large enterprises are significantly positive, the influences of cross-disciplinary background in small and large enterprises are significantly positive, and the marginal effects number of subordinates in the micro and small enterprises are significantly positive, while the job-hopping experience has significant positive effects in all different scales of the enterprises. In terms of career development, the coefficients of position promotion and relative income are significantly negative in small- and medium-sized enterprises, while the coefficient of skills upgrading in the micro and large enterprises is significantly negative. The empirical results show that for the HR in the small- and medium-sized enterprises; if they are easy to be promoted and get a satisfactory income level in this company, it will reduce their entrepreneurial intentions. As for the HR staff in micro and large enterprises, if they have certain opportunities to enhance their skills, their entrepreneurial intention will be correspondingly weakened.

**Table 5:** Heterogeneous analysis results for different enterprise scales.

**Dimension**	**Variables**	**(5) Different enterprise scales**			
		**Micro enterprises**	**Small enterprises**	**Medium enterprises**	**Large enterprises**
Entrepreneurial capability	Human Resource Management background	0.035 (0.075)	0.020 (0.033)	0.080^*^ (0.044)	0.094^*^ (0.057)
	Cross– discipline background	0.125 (0.083)	0.137^***^ (0.04)	0.082 (0.051)	0.152^***^ (0.057)
	Job–hopping experience	0.061^**^ (0.024)	0.024^*^ (0.013)	0.036^**^ (0.018)	0.069^***^ (0.020)
	Number of subordinates	0.071^**^ (0.03)	0.048^***^ (0.014)	0.018 (0.017)	−0.008 (0.016)
Development space	Position promotion	0.058 (0.040)	−0.063^***^ (0.019)	−0.051^**^ (0.025)	0.001 (0.030)
	Skills upgrading	−0.092^**^ (0.039)	−0.004 (0.019)	−0.026 (0.025)	−0.071^**^ (0.029)
	Relative income	−0.047 (0.063)	−0.099^***^ (0.027)	−0.104^***^ (0.038)	−0.028 (0.044)
Control variables		Yes	Yes	Yes	Yes
Wald χ^2^		64.34^***^	131.72^***^	100.33^***^	87.16^***^
Observations		184	1,018	570	447

The symbols ^***^, ^**^ and ^*^ are significant at the level of 1%, 5% and 10% respectively.

Table 6 shows that the effects of entrepreneurial capability and career development in different cities are also heterogeneous. From the perspective of entrepreneurial capability, in the first-tier cities, the coefficients of cross-disciplinary background, job-hopping experience, and the number of subordinates are significantly positive. In the new first-tier cities, the influences of HRM background, cross-disciplinary background, and job-hopping experience are significantly positive. In the second-tier cities, only the coefficient of cross-discipline is significantly positive. In the third-tier cities, there are obvious positive effects of HRM background, job-hopping experience, and the number of subordinates on the entrepreneurial intention. In terms of career development, lack of promotion space and relatively lower income are significantly positive in the first-tier and new first-tier cities. However, in the second- and third-tier cities, the impacts of career development variables are not obvious. The reason is that there are differences in career development opportunities in various cities. Compared with the second- and third-tier cities, the first-tier and new first-tier cities have more career development opportunities and large promotion space. If the HRs lack career development opportunities in their original enterprises, or if their actual income is lower than expected income, they prefer to leave the original enterprises and start their own business, and their entrepreneurial intentions will be stronger.

**Table 6:** Heterogeneous analysis results in different cities levels.

**Dimension**	**Variables**	**(6) Different cities levels**			
		**The first–tier** **cities**	**The new first–tier cities**	**The second-tier cities**	**The third-tier cities**
Entrepreneurial capability	HR management background	0.002 (0.030)	0.121^**^ (0.048)	0.016 (0.076)	0.107^*^ (0.062)
	Cross- discipline	0.117^***^ (0.032)	0.136^**^ (0.068)	0.227^**^ (0.093)	0.043 (0.081)
	Job-hopping	0.026^**^ (0.012)	0.064^***^ (0.019)	0.030 (0.032)	0.081^***^ (0.026)
	number of subordinates	0.022^**^ (0.011)	−0.036 (0.023)	0.021 (0.029)	0.059^***^ (0.020)
Career	Promotion	−0.029^*^ (0.017)	−0.052^*^ (0.029)	−0.033 (0.045)	−0.032 (0.036)
development	Skill improvement	−0.022 (0.017)	−0.028 (0.028)	−0.009 (0.045)	−0.047 (0.035)
	Relative income	−0.081^***^ (0.026)	−0.110^***^ (0.040)	0.011 (0.071)	−0.077 (0.047)
Control variables		Yes	Yes	Yes	Yes
Wald χ^2^		168.35^***^	91.44^***^	46.21^***^	74.35^***^
Observations		1,279	431	191	318

The symbols ^***^, ^**^ and ^*^ are significant at the level of 1%, 5% and 10% respectively.

## Discussion

This study investigates the effect of entrepreneurial capability and development space on entrepreneurial intention. First, we put forward corresponding research hypotheses. Based on 2018 China's Human Resource Employees Survey, we use the probit model to test the impacts of entrepreneurial ability and career development empirically. After that, we also further explore the heterogeneity results and make clear the impacts of entrepreneurial capability and career development in different enterprises, which involves in enterprises of different scales and different types. The empirical results show that entrepreneurial capability can effectively predict the entrepreneurial intention, while career development may have a negative impact on entrepreneurial intention. In addition, we also find that the results are heterogeneous in different city levels and enterprises of different scales and different types.

### Theoretical Contributions

Based on the background of “mass entrepreneurship and innovation,” this study expands and adds depth to the literature related to entrepreneurial ability, career development, and entrepreneurial intention in the following three aspects.

First, in view of the research perspective, existing studies have mainly discussed the new characteristics of entrepreneurship in the Internet era, entrepreneurial passions, and entrepreneurship education (Wu et al., 2018; Yang et al., 2021), and these studies mainly focus on the general entrepreneurs or graduate entrepreneurs and summarize the factors of entrepreneurial success (Wu et al., 2019; Yuan et al., 2020; Chen et al., 2021). Different from existing studies, which focus on the general entrepreneurs or graduate entrepreneurs, this study focuses on the specific group of HR staff and discusses the impact of entrepreneurial ability and career development on their entrepreneurial intention, which enriches the research in related fields.

Second, in terms of the research methods, qualitative research methods such as literature research, case analysis, and in-depth interview are often used in existing studies on entrepreneurial intention (Mair and Marti, 2009; Heilbrunn, 2010). However, due to the limited number of samples, this study constructed an econometric model and conducted a quantitative analysis on 2,219 samples of HR staff and uses descriptive statistics, least square regression, and subsample regression to test the impact of entrepreneurial ability and career development on HR entrepreneurial intention, and it discusses the relationship between variables in a quantitative way.

Third, in terms of research contents, unlike the existing literature, which analyzes entrepreneurs as homogeneous groups (Katila et al., 2021; Yurrebaso et al., 2021), this study discusses the heterogeneity of the entrepreneurial intentions for different HR staff and makes a subsample analysis according to the enterprise type, enterprise scale, and city level, and it investigates the heterogeneity of influencing factors for entrepreneurial intentions in different enterprise.

### Practical Implications

Our analysis has some implications for entrepreneurship. The development of the digital economy and the platform employment has provided new opportunities for HR groups to start entrepreneurship. Especially in the context of “three-pillar human resource,” HR has seen opportunities for entrepreneurship in the fields of Human Resource Business Partner (HRBP), Center of Expertise (COE), and Shared Service Center (SSC). This study focuses on the groups of HR staff, and it analyzes the impact of entrepreneurial ability and career development on HR's entrepreneurial intention and puts forward the corresponding research enlightenment.

First, entrepreneurial ability should be regarded as an important starting point for the success of entrepreneurship. HR's professional background, cross-disciplinary knowledge, job-hopping experience, and the number of subordinates constitute HR's entrepreneurial ability, and these can effectively predict their entrepreneurial intention. Different from traditional employment, entrepreneurship involves professional skills, multiple knowledge structures, and leadership ability, which requires a higher comprehensive ability of entrepreneurs, hence HR entrepreneurs should belong to the interdisciplinary talent, who not only require the knowledge of HRM but also are familiar with other disciplinary knowledge. At the same time, they must also have the rich practical experience and leadership skills; these entrepreneurial abilities help strengthen HR's entrepreneurial intention.

Second, it is necessary to improve the career development system to create more on-the-job entrepreneurial opportunities for HR. This study shows that if HR has many opportunities for job promotion and skill upgrading or the actual income exceeds the expected value, it will have a high cost to quit the current job, which may hinder their entrepreneurial intention. This article suggested that we should attach importance to the influence of career development on entrepreneurial intention. On the one hand, we should create HR's career development system and institutional environment and encourage HR to carry out on-the-job entrepreneurship through project cooperation, talent selection, and business outsourcing. On the other hand, part-time entrepreneurs should enjoy the appropriate rights to participate in job promotion and project application, and these practices help dispel concerns about part-time entrepreneurship.

Third, different and precise entrepreneurial support policies should be implemented for HR in different enterprises. Combined with the conclusions of this study, it is revealed that for HR in different enterprise types, enterprise sizes, and city levels, the impacts of entrepreneurial ability and career development on their entrepreneurial intention are heterogeneous. For HR employees in public institutions, state-owned enterprises, large-sized enterprises, and first-tier cities, due to their low mobility and high turnover costs, we should encourage them to participate in entrepreneurship by improving the talent flowing system and establishing on-the-job entrepreneurship guarantee policies. For HR employees in foreign enterprises, private enterprises, small- and medium-sized enterprises, second- and third-tier cities, their mobilities are strong, but they have shortcomings in theoretical knowledge and working experience. In the future, we should strengthen the cultivation of their entrepreneurial ability to improve their entrepreneurship intention.

## Limitations and Development

This study preliminarily explores the influence of entrepreneurial ability and career development on HR's entrepreneurial intention, and there are some issues that still need to be further discussed. In terms of data, this study uses micro-survey data, which focuses on HR groups; although the questionnaire involves entrepreneurship intention, career development, and other related issues, there are certain limitations in the research sample, and we suggest expanding the sample groups in the future research. In terms of research issues, this study focuses on entrepreneurial intention, because the entrepreneurial intention is the main reason for determining entrepreneurial actions, and further research should be conducted on entrepreneurial action. In terms of research methods, this study uses the probit model to analyze the influencing factors of HR's entrepreneurial intention. In the future, panel data models, structural equations, and other methods can be used to carry out relevant studies.

## Data Availability Statement

The original contributions presented in the study are included in the article/supplementary material, further inquiries can be directed to the corresponding author/s.

## Ethics Statement

Ethical review and approval were not required for the study on human participants in accordance with the local legislation and institutional requirements. The patients/participants provided their written informed consent to participate in this study.

## Author Contributions

WXH designed the theoretical model and wrote the original manuscript. HL and YFM analyzed the data and improved the manuscript. XHZ contributed to writing review and editing. YQT revised and supervised the entire work. All authors contributed to the article and approved the submitted version.

## Funding

This research was supported by the Youth Program of Beijing Social Science Foundation (21JJC022).

## Conflict of Interest

The authors declare that the research was conducted in the absence of any commercial or financial relationships that could be construed as a potential conflict of interest.

## Publisher's Note

All claims expressed in this article are solely those of the authors and do not necessarily represent those of their affiliated organizations, or those of the publisher, the editors and the reviewers. Any product that may be evaluated in this article, or claim that may be made by its manufacturer, is not guaranteed or endorsed by the publisher.
